# Supplementary material for: The impact of cross-reactive immunity on the emergence of SARS-CoV-2 variants
Source: Front Immunol. 2023 Jan 11;13:1049458. doi: 10.3389/fimmu.2022.1049458 (PMC9874934; doi:10.3389/fimmu.2022.1049458)
Supplement: Supplementary file 1 [file DataSheet_1.pdf]

Supplementary Information for "The impact of cross-reactive immunity on the emergence of SARS-CoV-2 variants"

R.N. Thompson, E. Southall, Y. Daon, F.A. Lovell-Read, S. Iwami, C.P. Thompson, U. Obolski

Text S1. Stochastic simulations

In Section 2.1 of the main text, we present a deterministic model that describes the transmission dynamics of a novel variant (system of equations (2) in the main text). This model accounts for cross-reactive immunity due to the earlier spread of an antigenically related virus. However, since we are considering the ability of the novel variant to invade the host population, it is necessary for us to instead use a stochastic model in which the novel variant can either invade the host population or fade out. Here, we describe the stochastic model that we use (this model is the stochastic analogue to the deterministic model shown in system of equations (2) in the main text).

As in system of equations (2) in the main text, individuals are split into compartments. Specifically, S_n, E_n, I_n and R_n reflect the infection status (Susceptible, Exposed, Infectious and Removed, respectively) with the novel variant of individuals who have not been infected previously by the related virus. The compartments S_p, E_p, I_p and R_p refer to the infection status with the novel variant of individuals who have previously been infected by the related virus. In the model, the transitions between compartments below are possible and occur at the following rates (where events refer to the novel variant; for example, an infection event refers to an individual being infected by the novel variant):

**Event**	**Rate**
Infection of a susceptible individual who was not previously infected by the related virus ($S_n \rightarrow S_n - 1$, $E_n \rightarrow E_n + 1$)	$\beta I_n S_n + \beta(1-\varepsilon) I_p S_n$
Onset of infectiousness of an exposed individual who was not previously infected by the related virus ($E_n \rightarrow E_n - 1$, $I_n \rightarrow I_n + 1$)	γE_n
Removal/recovery of an infectious individual who was not previously infected by the related virus ($I_n \rightarrow I_n - 1$, $R_n \rightarrow R_n + 1$)	μI_n
Infection of a susceptible individual who was previously infected by the related virus ($S_p \rightarrow S_p - 1$, $E_p \rightarrow E_p + 1$)	$\beta(1-\alpha) I_n S_p + \beta(1-\varepsilon)(1-\alpha) I_p S_p$
Onset of infectiousness of an exposed individual who was previously infected by the related virus ($E_p \rightarrow E_p - 1$, $I_p \rightarrow I_p + 1$)	γE_p
Removal/recovery of an infectious individual who was previously infected by the related virus ($I_p \rightarrow I_p - 1$, $R_p \rightarrow R_p + 1$)	μI_p

This model is simulated using the direct method version of the Gillespie stochastic simulation algorithm [1]. Specifically, the following steps are followed:

1. Initialise the numbers of individuals in each of the compartments of the model (S_n, E_n, I_n, R_n, S_p, E_p, I_p and R_p) and set the outbreak time *t* = 0.
2. Steps 2-4 are repeated while the outbreak is still ongoing (i.e. $E_n + I_n + E_p + I_p > 0$). First calculate two random numbers r_1, r_2 each uniformly distributed in (0,1).
3. Calculate the time of the next event by sampling from an exponential distribution. Set
$$t = t + \frac{1}{\lambda} \ln\left(\frac{1}{r_1}\right),$$
where λ is total rate at which events are occurring (i.e. the sum of the second column in the table above).
4. Choose the type of the next event. Determine which of the following statements is true:
- If $r_2 < \frac{\beta I_n S_n + \beta(1-\varepsilon) I_p S_n}{\lambda}$, then the next event is the infection of a susceptible individual who was not previously infected by the related virus: Set $S_n = S_n - 1$ and $E_n = E_n + 1$.

- If $\frac{\beta I_n S_n+\beta(1-\varepsilon) I_p S_n}{\lambda} \leq r_2 < \frac{\beta I_n S_n+\beta(1-\varepsilon) I_p S_n+\gamma E_n}{\lambda}$, then the next event is the onset of infectiousness of an exposed individual who was not previously infected by the related virus: Set $E_n = E_n - 1$ and $I_n = I_n + 1$.

- If $\frac{\beta I_n S_n+\beta(1-\varepsilon) I_p S_n+\gamma E_n}{\lambda} \leq r_2 < \frac{\beta I_n S_n+\beta(1-\varepsilon) I_p S_n+\gamma E_n+\mu I_n}{\lambda}$, then the next event is the removal/recovery of an infectious individual who was not previously infected by the related virus: Set $I_n = I_n - 1$ and $R_n = R_n + 1$.

- If $\frac{\beta I_n S_n+\beta(1-\varepsilon) I_p S_n+\gamma E_n+\mu I_n}{\lambda} \leq r_2 < \frac{\beta I_n S_n+\beta(1-\varepsilon) I_p S_n+\gamma E_n+\mu I_n+\beta(1-\alpha) I_n S_p+\beta(1-\varepsilon)(1-\alpha) I_p S_p}{\lambda}$, then the next event is the infection of a susceptible individual who was previously infected by the related virus: Set $S_p = S_p - 1$ and $E_p = E_p + 1$.

- If $\frac{I_n S_n+\beta(1-\varepsilon) I_p S_n+\gamma E_n+\mu I_n+\beta(1-\alpha) I_n S_p+\beta(1-\varepsilon)(1-\alpha) I_p S_p}{\lambda} \leq r_2 <$ $\frac{\beta I_n S_n+\beta(1-\varepsilon) I_p S_n+\gamma E_n+\mu I_n+\beta(1-\alpha) I_n S_p+\beta(1-\varepsilon)(1-\alpha) I_p S_p+\gamma E_p}{\lambda}$, then the next event is the onset of infectiousness of an exposed individual who was previously infected by the related virus: Set $E_p = E_p - 1$ and $I_p = I_p + 1$.

- If $r_2 \geq \frac{I_n S_n+\beta(1-\varepsilon) I_p S_n+\gamma E_n+\mu I_n+\beta(1-\alpha) I_n S_p+\beta(1-\varepsilon)(1-\alpha) I_p S_p+\gamma E_p}{\lambda}$, then the next event is the removal/recovery of an infectious individual who was previously infected by the related virus: Set $I_p = I_p - 1$ and $R_p = R_p + 1$.

References

1. **Gillespie DT. 1977.** Exact stochastic simulation of coupled chemical reactions. *J Phys Chem* **81**: 2340-2361.

Supplementary figures

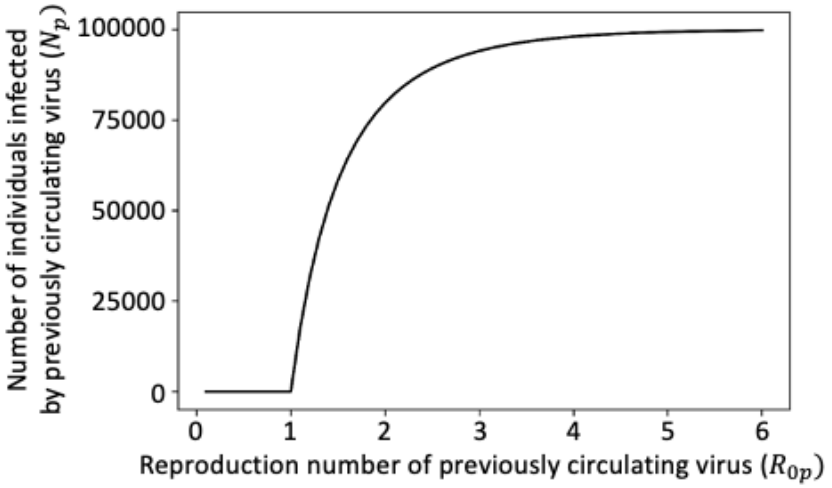

Figure S1. Dependence of the number of individuals infected by the previously circulating virus (N_p) on the reproduction number of the previously circulating virus accounting for interventions (R_{0p}). The value of N_p is calculated for each value of R_{0p} by solving equation (1) in the main text numerically, assuming a population of size $N = 100{,}000$.

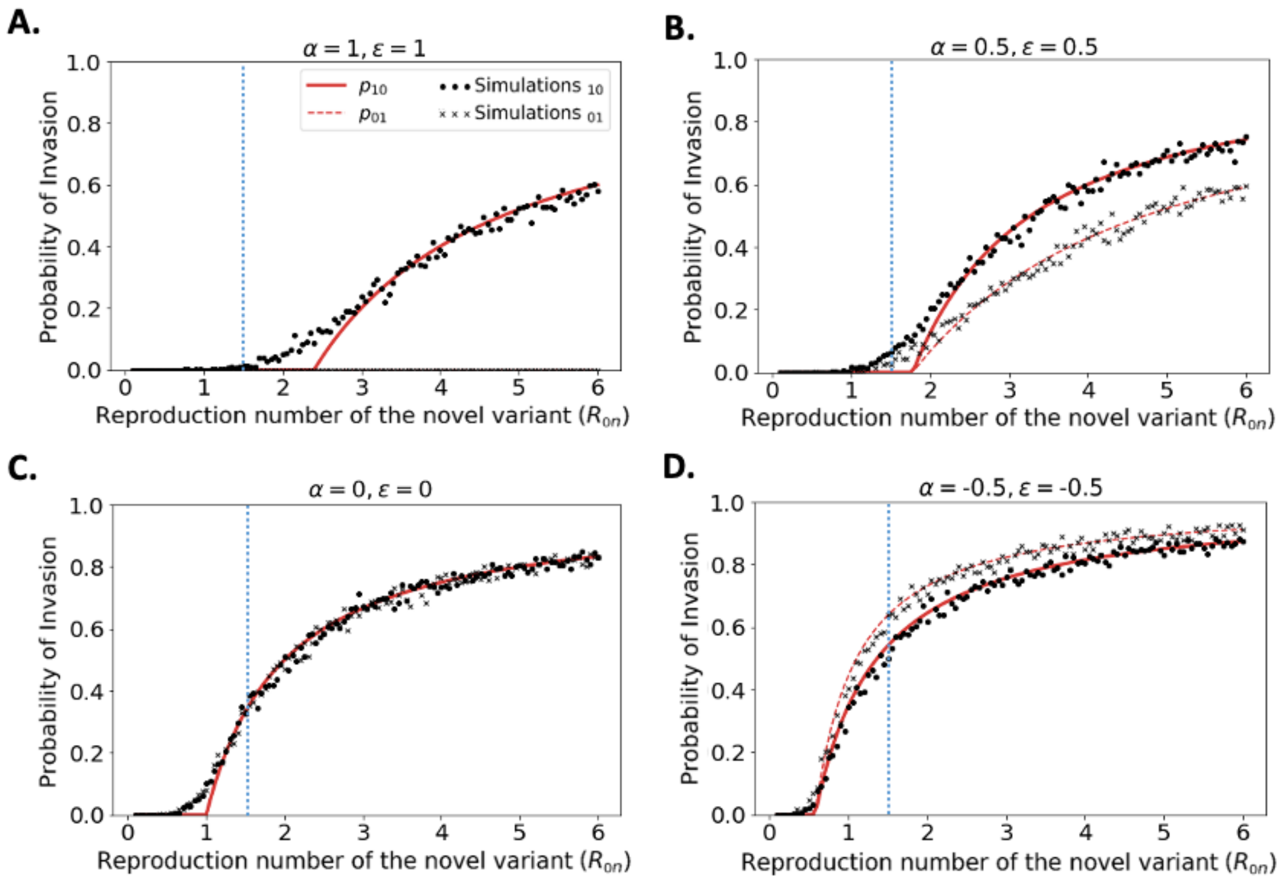

Figure S2. Probability of the novel variant invading the host population, starting from the introduction of a single infectious individual. Results analogous to Fig 2 of the main text, except that in the simulations here the probability of invasion was calculated as the proportion of simulations in which the number of simultaneously infected individuals ($I_n + I_p$) exceeded 5 at any time (rather than 15 as in Fig 2 in the main text).

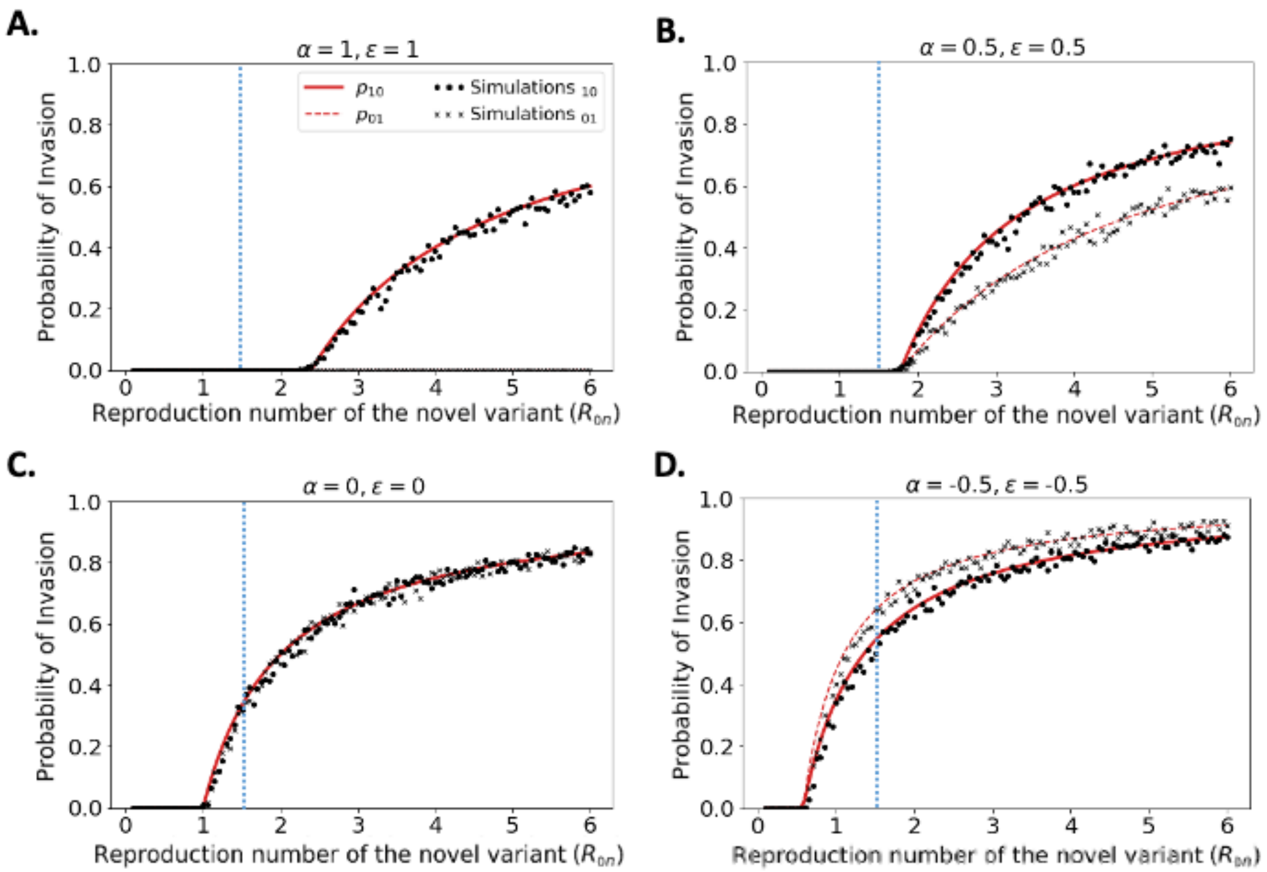

Figure S3. Probability of the novel variant invading the host population, starting from the introduction of a single infectious individual. Results analogous to Fig 2 of the main text, except that in the simulations here the probability of invasion was calculated as the proportion of simulations in which the number of simultaneously infected individuals ($I_n + I_p$) exceeded 30 at any time (rather than 15 as in Fig 2 in the main text).

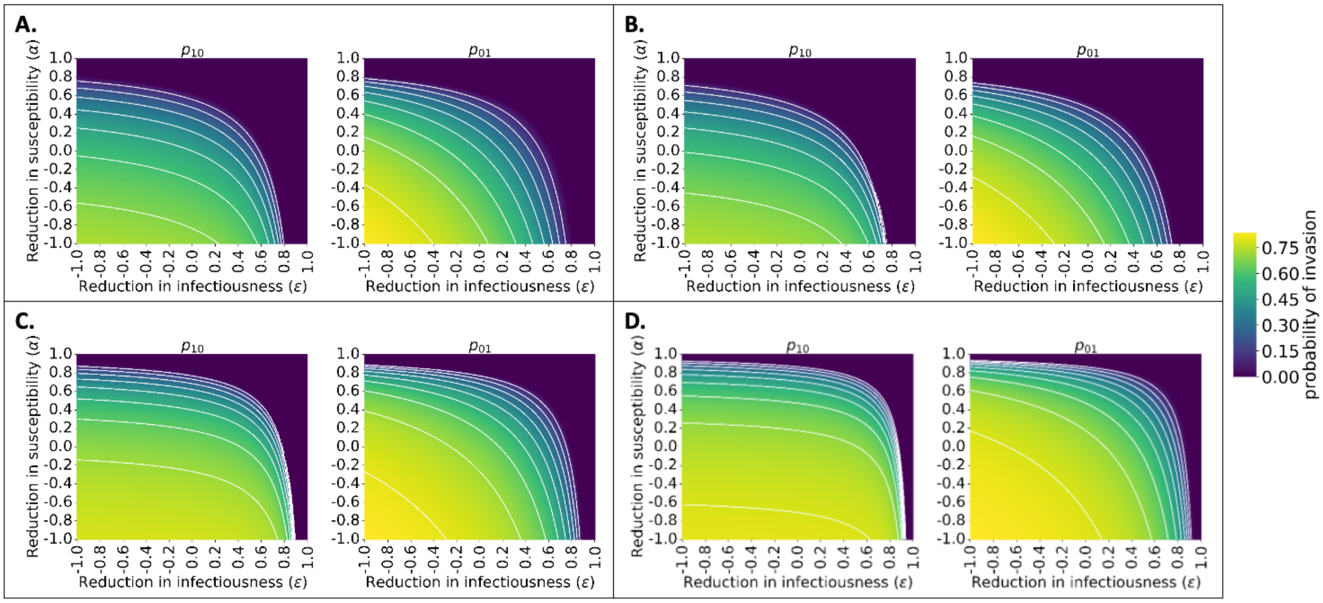

Figure S4. Probability of the novel variant invading the host population, starting from the introduction of a single infectious individual, for different levels of cross-reactive immunity affecting susceptibility and infectiousness individually. Results analogous to Fig 3 of the main text, but for different values of R_{0p} and R_{0n}, specifically: A. $R_{0p} = 2$, $R_{0p} = 2$; B. $R_{0p} = 3$, $R_{0n} = 2$; C. $R_{0p} = 3$, $R_{0n} = 4$; D. $R_{0p} = 4$, $R_{0n} = 6$.
